# Mechanical Abrasion of the Teeth

**Published:** 1872-09

**Authors:** S. H. Guilford


### ARTICLE II.

### *Mechanical Abrasion of the Teeth.*

BY S. H. GUILFORD, D. D. S.

Read before the Pennsylvania State Dental Society.

At the present time, when the profession of our choice is aspiring to and rapidly attaining that place among the learned professions which it is destined at no very distant day to occupy, the minds of its members are constantly employed in developing things already known, and in seeking out new facts which, when properly brought to light and developed, shall assist in raising her to her justly deserved position.

We have those minds among us which are particularly calculated to give birth to new ideas, and also assist in their development; and, on the other hand, we have those who, although not given to the propagation of new ideas, are specially gifted in apprehending them, weighing their value, and developing any good qualities they may possess; provided they possess any at all.

These two opposites, in reality component parts, are just what we need to develop our beloved profession. Neither is of value alone, and each is invaluable when joined.

While not arrogating to ourselves membership in the first class, we hope we may freely be accorded fellowship with the second, and hence to-day, if we treat you with nothing new, we hope to try to develop the old.

Amid the ever varying subjects which have engrossed the dental mind in times past, and even until now, we think that childhood, youth and middle age have received, as they have indeed deserved, a fair share of our attention. First and second dentition have, from the beginning, been favorite themes for the author and lecturer, whilst the treatment of irregularities and filling of teeth have been among the honored subjects of society essayists and magazine writers.

It is, in our opinion, past middle and old age, which, if any, have been lacking in that attention which they have a proper right to expect from us, as laborers in the field.

As far as our observation and experience goes, it has been entirely too common for us, while devoting our best energies of mind and body to childhood and middle age, to think that the only attention the teeth of past, middle or old age deserves at our hands is extraction and replacement. Older persons, it is true, are sometimes more remiss in their attention to their teeth than they should be, and thus permanently induce or at least give license to decay. But it is generally done through the mistaken idea that the time has about come when all lose their natural teeth, and hence there is no longer any use in trying to save them. It is one of our highest duties as dentists, in the proper signification of that term, to correct this wrong impression, and to show by our words and actions that there is hope even for the elderly —that they may oftentimes save and use for years longer their natural teeth, instead of supplanting them with artificial ones, nature's great inferiors.

A thoughtful consideration of these facts has resulted in our deciding to offer you a few remarks, this morning, upon the "Mechanical Abrasion of the Teeth, and its Remedies."

This mechanical abrasion, as you all know, is a wearing away of the substance of the natural teeth on their masticating surfaces, and is seldom, perhaps never, seen except in persons who have arrived at that period of life when nature has ceased to fully repair the loss of wear and tear. Earlier in life, when the vital forces are in their prime, enabling nature from the abundance of her store-house to replace all that is taken from her, this waste is seldom noticed; but later in life, when her energies begin to fail, and the waste is as great as ever, and perhaps greater, she begins gradually to lose ground and yield, though very reluctantly, the field to her enemy. This peculiar kind of abrasion is by far more frequently seen where the front teeth meet edge to edge instead of over-lapping each other as they should, although it is sometimes seen where the teeth have a perfectly natural occlusion. The two models which I have brought with me, for your inspection, are representatives of both classes, one of each.

Although this principle of waste exceeding repair after a certain period of life (and which is a universal law, noticeable throughout the whole animal economy,) and the meeting of the teeth edge to edge, are the principal causes of this abrasion, still they are generally assisted in their work of destruction by some other causes.

One cause is the loss of some of the bicuspids and molars of one or both jaws, thus throwing the great strain and burden of mastication on the incisors and cuspidati, and thereby giving them often double and triple the amount of work to perform that properly belongs to them; and another cause is the use of chewing tobacco, which most persons who use it keep constantly under the process of mastication; and worse than that, in its destructive effects, is the grit or fine sand, always found present in a greater or less degree in every variety of tobacco prepared for this filthy use.

This grit, under almost constant attrition, will, as a matter of course, soon wear away a considerable portion of the tooth substance, and it is in the mouths of users of this vile weed that we have noticed this form of abrasion in its greatest extent. The effects of this mechanical abrasion present themselves from time to time to the eye of the dentist in every variety and degree of extent.

Oftentimes, when this condition has but just commenced, the effects are, perhaps, only the wearing off of the sharp points of the cuspids and bicuspids, or the straightening or flattening of the edges of the incisors; and again in others, where the teeth have been unprotected for a longer time, we occasionally see the six front teeth literally worn down to the gums.

At first, while the enamel only is being destroyed, no ill effects or inconvenience are felt; but when it once reaches the dentine, or nearly approaches the pulp chamber, the inconvenience and pain resulting therefrom are greatly noticed and much complained of, and it is then most frequently that we are for the first time applied to for relief.

Frequently, indeed, in the great majority of cases as this destruction goes on and nears the vital portion of the tooth, nature in a measure protects herself by throwing up her bulwark of defense—osteo-dentine, but, eventually, as age advances, if the cause continues and no help is furnished, she has to succumb to her enemy, and the pulp becomes nearly or quite exposed. Serious as this evil is, and calling as it loudly does for relief at our hands, we do not find it even alluded to in any of the dental works that we have consulted, except in Harris, and he only devotes one page to it, hurriedly describing its characteristics and effects, and suggesting no relief or remedy whatsoever.

Whether the cases coming under his and other authors' notice were but limited in extent, or whether they considered them too insignificant to occupy a place in their works, we cannot tell, yet one thing we do know, and that is, that this disease, if we may so term it, is prevailing largely all over the country, particularly in the rural districts, and it is time more notice should be taken of it and the question of its remedy be more generally discussed.

What means of relief shall we prescribe and administer when appealed to by sufferers from this calamity? There seems to us to be but three plausible and practicable ways in which we can bring relief to the patient:

First—Is the old and probably by far the most universally common way of destroying the pulps of such teeth as fast as they become exposed and troublesome, and then filling the canals with gold. One argument in favor of this treatment is, that it brings immediate relief to the patient; and another is, that it is probably the least expensive of all operations for permanent relief. The objections to it, however, are, that it does not prevent the future wearing away of the teeth, but rather, indeed, by loss of nourishment promotes it, and, also, half a dozen or more lifeless teeth in the mouth are never as desirable as so many living ones.

Another operation for relief consists in building up the crowns of the abraded teeth to their original normal shape and size. This is done by drilling a number of retaining pits or points in the dentine between the enamel and pulp, and from these by the aid of adhesive foil, and perhaps the mallet, building down the golden crowns. The first record of such an operation, within our recollection, is the one performed by Dr. Atkinson, of New York, for a gentleman from Sacramento, California, as described and illustrated in the January number of the *Dental Cosmos* for 1864.

An operation of this kind on some half dozen teeth well done, and intended to perform henceforth a large portion of the mastication of the individual, is certainly in the very highest style of art, and establishes immediately the reputation of him who performs it as a master in the art of filling. It also is praiseworthy, in that it restores the teeth to their original shape. Though this style of work may commend itself to some few in the larger cities, there are objections to it which will, we think, prevent it from becoming the commonly accepted mode of dealing in such cases.

First of all, as it requires the best skill, it will also command a very high price, which many would be unwilling to pay, and in the rural districts few would really be able to, especially if they were presented with bills such as our California friend was.

Next comes the objection of unsightly appearance. We approve of the building up of a single tooth or portion of one, lost through disease or accident, and have frequently performed the operation ; but to see a row of teeth half gold or more, where the teeth were alike in appearance without it, does not commend itself to our taste, and we think there are many who would likewise object to the appearance of it.

Another objection to these operations is, that if these crowns are built down with foil, and closely fashioned to their original shape the incisors must necessarily be rather thin at their cutting edges, and if they overlap the inferior incisors, as in many cases the original teeth did, we do not believe that they will be able to bear the strain for many years to which they will necessarily be subjected in mastication. If the teeth originally occluded edge to edge, and in restoring them with gold they be built down but part way, and left with rather broad tops, the strain of mastica tion being exerted in a direct line with the axis of the tooth, I believe they may endure almost any length of time and do good service.

There is, however, another way of administering relief to patients of this class, which, we think, possesses more advantages and is open to fewer objections than either of the previously mentioned methods.

It consists in the making and introduction of a plate of gold or silver of suitable thickness, covering a portion of the roof of the mouth, retained in position by clasps where practicable, and accurately fitting into and covering the concavities of the abraded teeth. To construct the plate, an impression is taken in the usual way, with plaster where it can be, and where this cannot well be done, an impression taken in wax will do.

A model is then made, after which the die and counter-die are prepared, and the plate with frequent annealings, is swaged home to its shape. The plate, when swaged or while swaging, should be trimmed up to the exact shape of the outer margins of the teeth, and where these do not quite touch each other, the plate should be cut away between the teeth. It is usually swaged from a single piece of plate, and when the metal is firm and homogeneous in its character, there is little difficulty attending the process. To make the plate invisible, as it should be, it must be beveled to a knife edge at the anterior or buccal edge of each tooth.

If on trying it in the mouth it is found to touch too much at one place, or too little at others, as will often be the case, matters must be adjusted by filing off in one case, or by the soldering on of an extra thickness of plate at various places in the other.

When, in the course of time, the plate becomes too much worn at one place or another, it is easily remedied by flowing a little solder on the worn portion, or by adding an extra piece of thin plate.

Sometimes it is desirable to add a few artificial teeth to your capping-plate to supply the loss of natural ones. In such an event the plate is swaged, clasps attached, and the bite adjusted, as previously described, and when all is satisfactory the teeth can be added as in ordinary cases.

The advantages of protecting abraded teeth by such a plate are, that the sensitiveness of the teeth to heat, cold, acids, etc., is entirely overcome as soon as the plate is introduced, because covered securely by the metal. Again, there is no unsightly show of gold; in fact nothing whatever is seen if the plate has been properly constructed. The clasps retain it firmly in place under all circumstances, and admit of its ready removal for cleansing and subsequent replacement. When constant use has worn away any part of it, it can be repaired, if there be no artificial teeth upon it, in a few minutes. It can be constructed cheaply, compared with fillings, and with very little trouble. It is strong and durable, thoroughly reliable at all times, and will give general satisfaction.

In all my course of dental study, in college lectures and text books, and in all the periodical dental literature that I have read, I have never heard such capping plates spoken of or alluded to. The first idea in regard to them I received was learning incidently that a man had worn such a plate made of silver, and though clumsily constructed and badly fitting, yet it occurred to me that the idea was a good one, and that if properly developed and a gold plate of the same kind were neatly made and accurately fitted, it would be very serviceable in such a case.

I proceeded to make one for a patient in the manner described to you, and I am free to confess that the success that attended my efforts quite exceeded my own expectations.

I have brought a model of his mouth with me. The occlusion of his teeth, as you see, was edge to edge, and more singular than all was the fact that, although eight or ten of his anterior teeth were worn off almost to the gum, he had all his bicuspids and molars above and below in his mouth, and meeting each other in the most perfect manner. The other model beside it is one in which the occlusion was natural, and the abrasion consequently occurred on the lingual surface of the teeth, thus allowing their natural external appearance to remain unchanged. A gold plate was made for him, and he is now wearing it with the same satisfaction experienced by the patient first alluded to.

The little experience I have had in the construction of such plates, and the perfect satisfaction they have given in each instance, has led me to believe that they are just what is needed to prevent the further mechanical abrasion of the natural teeth, where it has already commenced, and it is hoped that the profession generally will take up the idea, give it a fair trial and then render a just verdict.

---

### ARTICLE III.

*Appeal in Behalf of the Wells National Testimonial Fund.*

